# A paediatric case of severe tracheobronchial injury successfully treated surgically after early CT diagnosis and ECMO safely performed in the hybrid emergency room

**DOI:** 10.1186/s13049-019-0628-0

**Published:** 2019-04-23

**Authors:** Daiki Wada, Koichi Hayakawa, Shuhei Maruyama, Fukuki Saito, Hiroyuki Kaneda, Yasushi Nakamori, Yasuyuki Kuwagata

**Affiliations:** 1 Department of Emergency and Critical Care Medicine, Kansai Medical University Medical Center, 10-15 Fumizono-cho, Moriguchi, Osaka, 570-8507 Japan; 2 Department of Thoracic Surgery, Kansai Medical University Medical Center, 10-15 Fumizono-cho, Moriguchi, Osaka, 570-8507 Japan; 3 Department of Emergency and Critical Care Medicine, Kansai Medical University Hospital, 2-3-1 Shinmachi, Hirakata, Osaka, 573-1191 Japan

**Keywords:** Tracheobronchial injury, Veno-venous extracorporeal membrane oxygenation (VV ECMO), Hybrid ER

## Abstract

**Background:**

In paediatric trauma patients, tracheobronchial injury can be a rare, life-threatening trauma. In 2011, we instituted a new trauma workflow concept called the hybrid emergency room (Hybrid ER) that combines a sliding CT scanning system with interventional radiology features to permit CT examination and emergency therapeutic intervention without moving the patient. Extracorporeal membrane oxygenation (ECMO) can lead to cannula-related complications. However, procedures supported by moveable C-arm fluoroscopy and ultrasonography equipment can be performed soon after early CT examination. We report a paediatric patient with tracheobronchial injury diagnosed by CT examination who underwent rapid resuscitation and safe installation of veno-venous (VV) ECMO in our Hybrid ER and was successfully treated by surgery.

**Case presentation:**

A 11-year-old boy was admitted to our Hybrid ER suffering blunt chest trauma. His vital signs were unstable with low oxygen saturation. Early CT examination was performed without relocation. CT revealed bilateral hemopneumothorax, bilateral lung contusion, left multiple rib fractures, and right bronchus intermedius injury. Because his oxygenation was severely low with a PaO_2_/FiO_2_ ratio (P/F) of 109, he was at very high risk during transport to the operating room and changing to one-lung ventilation. Thus, we established VV ECMO in the Hybrid ER before we performed thoracotomy under left lung ventilation in the operating room. After the P/F ratio improved, he was transferred to the operating room under VV ECMO. We performed middle- and lower-lobe resection and sutured the stump of the right bronchus intermedius to treat the complete tear of this branch. After his respiratory function recovered, VV ECMO was removed on postoperative day 5. After in-patient rehabilitation, he was discharged home on postoperative day 68 without sequelae.

**Conclusions:**

It is feasible to perform VV ECMO in the Hybrid ER, but one case does not conclude it is safe. In this case, the blood oxygenation improved, but there are no evidence to support the safety of the procedure or the advantage of ECMO initiation in the Hybrid ER rather than in the operating room.

## Background

### Introduction of hybrid ER into trauma workflow

**Fig. 1:**
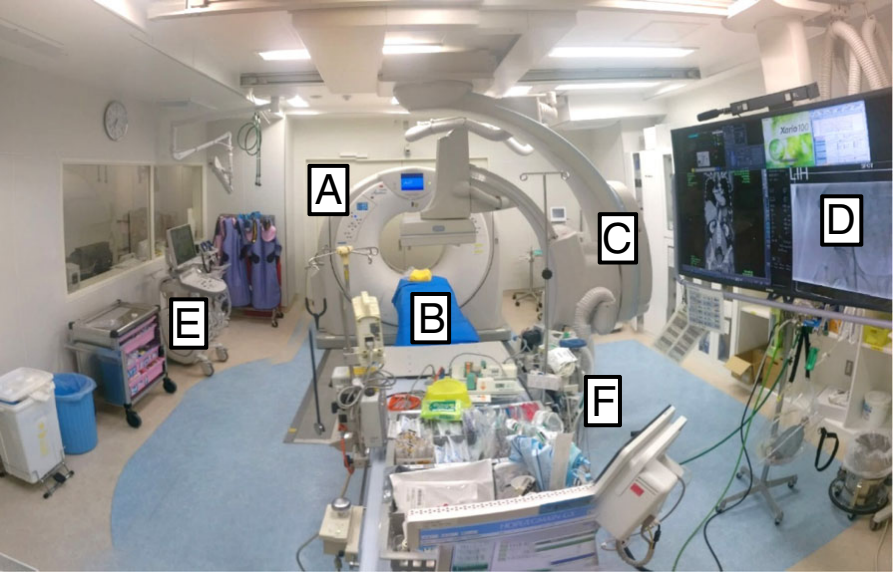
Photograph showing the Hybrid ER. All life-saving procedures including airway management, emergency surgery, and transcatheter arterial embolization can be performed on the procedure table without relocating the patient. The room contains **a** a sliding CT scanner, **b** CT examination and intervention table, **c** moveable C-arm, **d** 56-in. monitor screen, **e** ultrasonography equipment, and **f** a mechanical ventilator

In 2011, we instituted a new trauma workflow concept called the hybrid emergency room (Hybrid ER) that combines a sliding computed tomography (CT) scanner system with interventional radiology features to permit CT examination and emergency therapeutic interventions without moving the patient to another department (Fig. 1) [1]. A previous retrospective cohort study at a tertiary hospital showed that this novel trauma workflow allowing immediate CT diagnosis and rapid control of bleeding without transferring the patient to different departments may improve mortality in patients with severe trauma [2].

### The benefit of hybrid ER in the installation of ECMO

In severely injured patients and severely ill patients requiring percutaneous coronary intervention supported by veno-arterial or veno-venous extracorporeal membrane oxygenation (VV ECMO), the Hybrid ER is potentially beneficial because the cannula can be immediately and safely placed supported by moveable C-arm fluoroscopy and ultrasonography equipment soon after early CT examination.

### Tracheobronchial injury

Children rarely experience major tracheobronchial injury [3]. Because such rare injuries can cause life-threatening respiratory deficiency and high mortality, prompt diagnosis and efficient treatment have been shown to be major prognostic factors [3]. Urgent initiation of ECMO may be necessary before surgery if optimal chest drainage and ventilation fail to stabilise the patient [4]. We report a paediatric patient with tracheobronchial injury diagnosed by CT examination who underwent rapid resuscitation and the safe institution of VV ECMO in our Hybrid ER without relocating him and was successfully treated by surgery.

## Case presentation

**Fig. 2:**
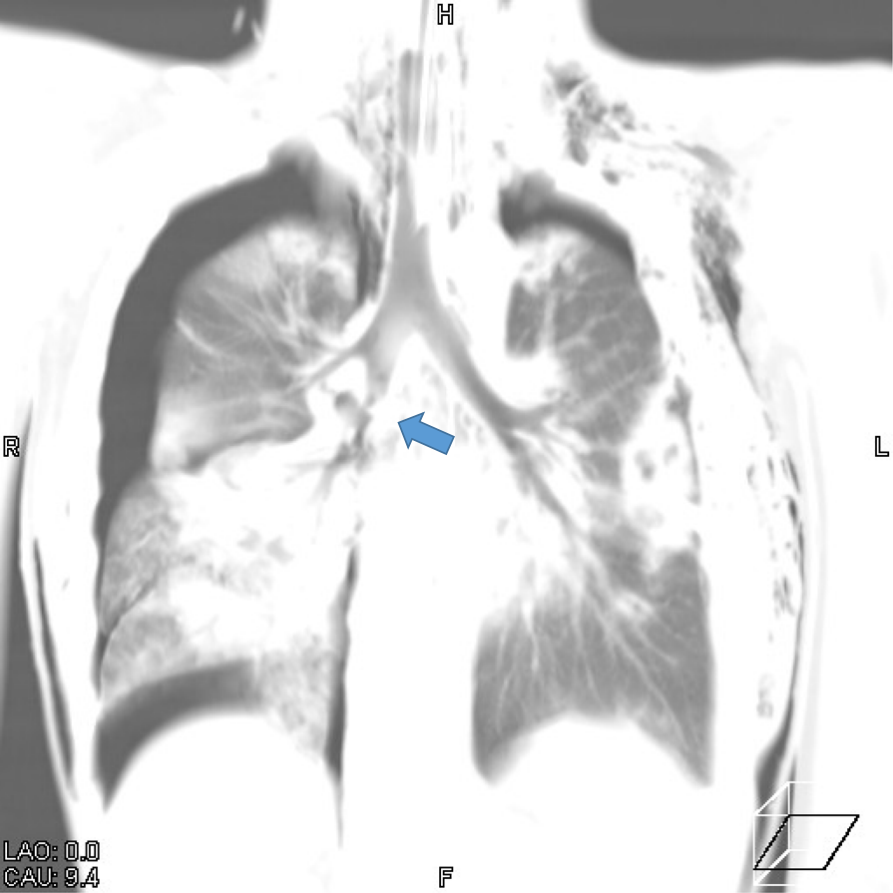
CT image shows right bronchus intermedius injury indicated by the blue arrow

An 11-year-old boy (weight, 35 kg; height, 154 cm) was admitted to our Hybrid ER with a history of blunt chest trauma after being run over by a truck. He was previously healthy, with no co-morbidities. During transport, the ambulance service administered oxygen at 10 L/min with a non-rebreather mask. The primary survey upon arrival to the emergency department: Airway (A) patent. Breathing (B) bilateral subcutaneous emphysema, right tension pneumothorax, tachypnea with a respiratory rate of 42/min and oxygen saturation of 66% on 10 L of oxygen by non-rebreather mask. Circulation (C) hemodynamically unstable with a pulse rate of 162/min and a blood pressure of 112/94 mmHg. Disability (D) Glasgow Coma Scale of 11/15. He was intubated with a single-lumen endotracheal tube, and intercostal drains were inserted on both sides of his chest for emergency pneumothorax. Arterial blood gas analysis performed under mechanical ventilation with a fraction of inspired oxygen (FiO2) of 1.0 showed a pH of 6.897, PaCO2 of 96.4 mmHg, PaO2 of 96.8 mmHg, BE of − 13.6 mmol/L, and lactate level of 74 mg/dL. After we perform these procedures, early CT examination was performed using the sliding CT scanner on the same trauma table without relocating him 20 min later from arrival. CT revealed the following injuries: bilateral hemopneumothorax, bilateral lung contusion, and left multiple rib fractures. Because the right chest tube revealed massive air leakage, we performed CT image processing by minimum intensity projection. As a result, we diagnosed right bronchus intermedius injury that required emergency thoracotomy (Fig. 2).

**Fig. 3:**
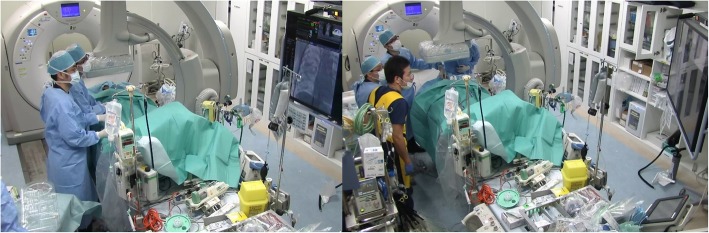
With the aid of the moveable C-arm in the Hybrid ER, a drainage cannula was placed in the patient’s right femoral vein and a return venous cannula was placed into his right internal jugular vein

**Fig. 4:**
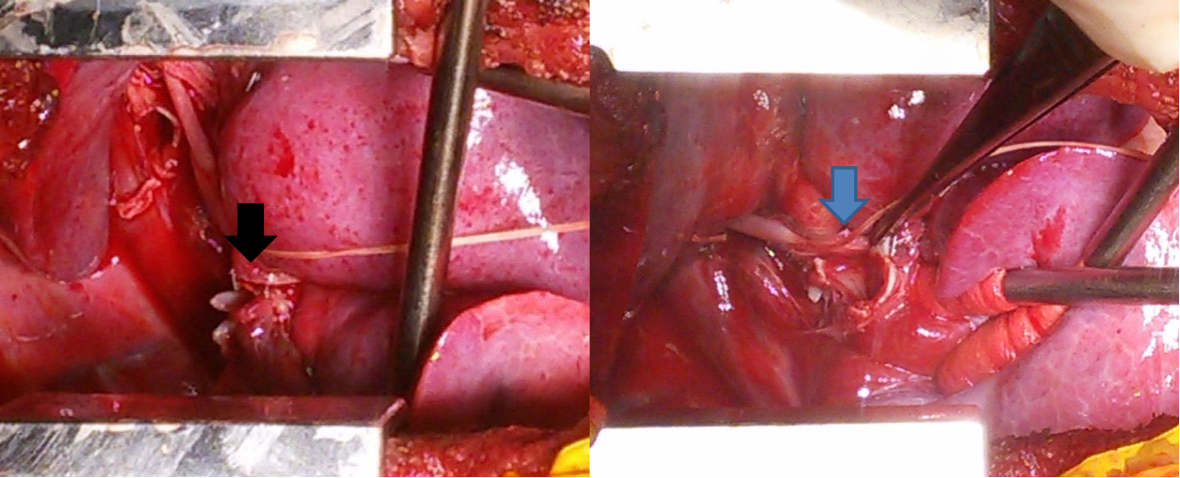
Intraoperative photographs show the complete tear of the right bronchus intermedius as indicated by the arrows. The black arrow indicates the proximal side and the blue arrow indicates the distal side

His oxygenation at this point was severely low with PaO2/FiO2 ratio (P/F) of 109. Considering the bilateral lung injury, he was at very high risk of low oxygenation during transport to the operating room and changing to left lung ventilation. We therefore decided to perform emergency thoracotomy under left lung ventilation in the operating room after our trauma team first stabilised his oxygenation with VV ECMO support in the Hybrid ER. Under moveable C-arm fluoroscopy and ultrasonography in the Hybrid ER, with percutaneous technique, a drainage cannula was placed in his right femoral vein and a return venous cannula was placed in his right internal jugular vein with the tips of both cannulae positioned in the right atrium (Fig. 3). Subsequently, the patient’s P/F ratio improved to more than 250, and he was transported to the operating room under VV ECMO. Anaesthesia was induced, and left lung ventilation was performed. Under VV ECMO support (ECMO flow of about 2 L/min; FiO_2_: 0.8), his oxygen saturation was maintained at over 98%. We did not use anticoagulants for the first 7 h. We started with a right lateral thoracotomy and found that there was a complete tear of the right bronchus intermedius (Fig. 4). To shorten the operating time, we performed middle lobe and lower lobe resection and sutured the stump of the right bronchus intermedius. To protect the ligated intermedius bronchus, we covered it with an intercostal muscle flap. During the operation for the tracheobronchial injury, left hemorrhagic pleural effusion increased at a rate of 200 ml in 1 h. Sequentially, we performed left anterolateral thoracotomy and performed surgical bleeding control for intercostal artery injury caused by the multiple left rib fractures. The operation required the transfusion of 12 units of blood and 6 units of fresh frozen plasma. After the surgical repair, the patient’s respiratory function recovered, and VV ECMO was removed on postoperative day 5. His Injury Severity Score was 26, Revised Trauma Score was 3.8, and the probability of survival by the Trauma and Injury Severity Score method was 59.45. Because of flail chest resulting from the multiple rib fractures, positive pressure ventilation was required for 3 weeks. He was completely weaned off mechanical ventilation on postoperative day 31. After in-patient rehabilitation, he was discharged home on postoperative day 68 without sequelae.

## Discussion

Although rare in the paediatric population, tracheobronchial rupture from blunt chest trauma can be a life-threatening injury [5]. Overall, tracheobronchial injury is rare among all age groups but is especially so in children although it is involved in up to 2.8% of severe blunt chest traumas resulting from road accidents deaths [6]. No specific guidelines have been published on the surgical treatment of tracheobronchial injury [7]. However, the general expert consensus is that emergency surgery for tracheobronchial injury should be performed when the esophageal wall prolapses into the tracheobronchial lumen, the patient cannot be ventilated properly, and tracheobronchial injury is found intraoperatively [8-11].

Injuries due to severe trauma are considered a contraindication for ECMO because of the probability of unstoppable bleeding [4]. ECMO is also used as postoperative supportive therapy in patients with severe pulmonary contusion or to prevent barotrauma after bronchial repair [4]. Recent reports have addressed adult patients with tracheobronchial injury requiring surgical repair with VV ECMO support [12]. Ryu and Chang reported the successful use of heparin-free ECMO in supporting an adult patient with tracheobronchial injury and pulmonary contusions prior to thoracotomy [13]. They revealed that, if a patient fails mechanical ventilator support because of tracheobronchial injury, advanced life support with ECMO may be required as a bridge to definitive surgical intervention.

To our knowledge, there are few paediatric cases of tracheobronchial injury requiring ECMO during emergency surgery. Ballouhey et al. reported on a 32-month-old girl in whom ECMO was used for ventilatory support during the life-saving surgical repair of a tracheobronchial injury [4]. Some cannula-related complication can occur in the establishment of ECMO, especially in paediatric patients. Ried et al. reported that cannula-related complications, mostly including ischemia or minor bleeding, occurred in 12% of trauma patients who underwent VV ECMO for extracorporeal pulmonary support [14]. Increasingly, the routine use of ultrasonographic guidance is being shown to be helpful in all types of vascular access. Banfi et al. suggested that ECMO cannulation can be achieved with use of ultrasonography and fluoroscopy in the cardiac catheterisation laboratory or in an operating room with C-arm fluoroscopy [15]. Fluoroscopy is essential in confirming proper positioning of the guidewires and cannulae [16]. In our pediatric case, considering the patient’s left lung hemopneumothorax and contusion, the use of only one-lung ventilation of the left lung might have led to severe hypoxemia before the emergency surgery. Thus, we safely initiated VV ECMO in the Hybrid ER via right femoral and right jugular cannulation under moveable C-arm fluoroscopy and ultrasonography after the CT examination without having to transfer the patient. After the operation was performed on the first day, there was no bleeding and thromboembolic complication during the VV ECMO support under the use anticoagulants.

We obtained a good outcome by introducing VV ECMO in the Hybrid ER before emergency thoracotomy in our patient with tracheobronchial injury. The initiation of VV ECMO in the Hybrid ER may be a safe and highly effective rescue treatment in a paediatric trauma patient with tracheobronchial injury. Even if under moveable C-arm and fluoroscopy the installation of ECMO was performed, we had some troubles as follows: (1) we could not get the enough blood flow from the drainage catheter by the catheter sticking to the wall of the vessel. (2) If we installed ECMO to the patient whose vessel was severely affected by arteriosclerosis, vascular injury sometimes occurred when we inserted the guidewire and catheter. We have not experienced enough number of trauma patients who required emergency VV ECMO to clarify the safeness and promptness of emergency VV ECMO introduced in the Hybrid ER. At present, 11 trauma centres have installed a Hybrid ER in Japan [17]. To further reveal the efficiency of our system in tracheobronchial injury, we need to collect and evaluate more cases in which definitive interventions with VV ECMO were performed.

## Conclusion

It is feasible to perform VV ECMO in the Hybrid ER, but one case does not conclude it is safe. In this case, the blood oxygenation improved, but there are no evidence to support the safety of the procedure or the advantage of ECMO initiation in the Hybrid ER rather than in the operating room.

## Abbreviations

CT: computed tomography
FiO_2_: fraction of inspired oxygen
Hybrid ER: hybrid emergency room
VV ECMO: veno-venous extracorporeal membrane oxygenation

## References

[1] Wada D, Nakamori Y, Yamakawa K, Fujimi S (2012). First clinical experience with IVR-CT system in the emergency room: positive impact on trauma workflow. Scand J Trauma Resusc Emerg Med.

[2] Kinoshita T, Yamakawa K, Matsuda H, Yoshikawa Y, Wada D, Hamasaki T (2019). The survival benefit of a novel trauma workflow that includes immediate whole-body computed tomography, surgery, and interventional radiology, all in one trauma resuscitation room: a retrospective historical control study. Ann Surg.

[3] Heldenberg E, Vishne TH, Pley M, Simansky D, Refaeli Y, Binun A (2005). Major bronchial trauma in the pediatric age group. World J Surg.

[4] Ballouhey Q, Fesseau R, Benouaich V, Léobon B (2013). Benefits of extracorporeal membrane oxygenation for major blunt tracheobronchial trauma in the paediatric age group. Eur J Cardiothorac Surg.

[5] Ballouhey Q, Fesseau R, Benouaich V, Lagarde S, Breinig S, Léobon B (2013). Management of blunt tracheobronchial trauma in the pediatric age group. Eur J Trauma Emerg Surg.

[6] Roxburgh JC (1987). Rupture of the tracheobronchial tree. Thorax.

[7] Grewal HS, Dangayach NS, Ahmad U, Ghosh S, Gildea T, Mehta AC. Treatment of tracheobronchial injuries: a contemporary review. Chest 2018. pii: S0012–3692(18)31112–7. doi: 10.1016/j.chest.2018.07.018. [Epub ahead of print]. 10.1016/j.chest.2018.07.018 PMC6435900 30059680

[8] Koletsis E, Prokakis C, Baltayiannis N, Apostolakis E, Chatzimichalis A, Dougenis D (2012). Surgical decision making in tracheobronchial injuries on the basis of clinical evidences and the injury's anatomical setting: a retrospective analysis. Injury.

[9] Deja M, Menk M, Heidenhain C, Spies CD, Heymann A, Weidemann H (2011). Strategies for diagnosis and treatment of iatrogenic tracheal ruptures. Minerva Anestesiol.

[10] Gabor S, Renner H, Pinter H, Sankin O, Maier A, Tomaselli F (2001). Indications for surgery in tracheobronchial ruptures. Eur J Cardiothorac Surg.

[11] Kaloud H, Smolle-Juettner FM, Prause G, List WF (1997). Iatrogenic ruptures of the tracheobronchial tree. Chest..

[12] Enomoto Y, Watanabe H, Nakao S, Matsuoka T (2011). Complete thoracic tracheal transection caused by blunt trauma. J Trauma.

[13] Ryu KM, Chang SW (2018). Heparin-free extracorporeal membrane oxygenation in a patient with severe pulmonary contusions and bronchial disruption. Clin Exp Emerg Med.

[14] Ried M, Bein T, Philipp A, Müller T, Graf B, Schmid C (2013). Extracorporeal lung support in trauma patients with severe chest injury and acute lung failure: a 10-year institutional experience. Crit Care.

[15] Banfi C, Pozzi M, Siegenthaler N, Brunner ME, Tassaux D, Obadia JF (2016). Veno-venous extracorporeal membrane oxygenation: cannulation techniques. J Thorac Dis.

[16] Shaheen A, Tanaka D, Cavarocchi NC, Hirose H (2016). Veno-venous extracorporeal membrane oxygenation (VV ECMO): indications, preprocedural considerations, and technique. J Card Surg.

[17] Watanabe H, Shimojo Y, Hira E, Kuramoto S, Muronoi T, Oka K (2018). First establishment of a new table-rotated-type hybrid emergency room system. Scand J Trauma Resusc Emerg Med..

